# Energy metric prediction for double insertion mutants via the RoseNet deep learning framework

**DOI:** 10.1093/bioadv/vbae198

**Published:** 2025-01-02

**Authors:** Sarah Coffland, Katie Christensen, Brian Hutchinson, Filip Jagodzinski

**Affiliations:** Computer Science Department, Western Washington University, Washington, 98225, United States; Computer Science Department, Western Washington University, Washington, 98225, United States; Computer Science Department, Western Washington University, Washington, 98225, United States; Joint Global Change Research Institute, Pacific Northwest National Laboratory, Maryland, 20740, United States; Computer Science Department, Western Washington University, Washington, 98225, United States

## Abstract

**Summary:**

Studying the structural and functional implications of protein mutations is an important task in computational biology and bioinformatics. We leverage our previously proposed RoseNet neural network architecture to predict energy metrics of proteins with double amino acid insertions or deletions (InDels). We train models on previously generated benchmark datasets containing the exhaustive double InDel mutations for three proteins, as well as an additional three proteins for which ∼145k random mutants, each with two InDels, have been generated. We expand on our previous work by evaluating three additional proteins and analyzing domain features that impact the prediction capabilities of RoseNet. These features include InDels into secondary structures and the solvent accessible surface area (SASA) scores of the residues. We uncover further evidence to support that RoseNet has a higher proficiency of generalizing to unseen residue combinations than unseen insertion positions. We also observe that RoseNet produces higher-quality predictions when inserting into a β-sheet over an α-helix. Additionally, when the insertions fall in an area of high SASA, RoseNet often displays better performance than inserting into areas of low SASA.

**Availability and implementation:**

The code used for training and evaluating the models in the study and the data underlying this article are available at https://github.com/hutchresearch/RoseNet.

## 1 Introduction

Machine learning techniques are gaining prominence in the fields of computational biology and bioinformatics. In areas such as drug discovery (Chebanov *et al.* 2023) and neuroscience (Stripelis *et al.* 2021), machine and deep learning approaches are seamlessly integrating into research practices. One facet in particular that is rapidly advancing through leveraging machine learning methods is proteomics (Li *et al.* 2020; Hie *et al.* 2024; Kozlova *et al.* 2023; Peng *et al.* 2023). Because the function of a protein is determined by its structure, the aim is to predict the functions of new or unseen protein mutants based on patterns found in preexisting protein structures (Alberts *et al.* 2002). Traditional wet lab techniques, including X-ray crystallography (Zalewski *et al.* 2017), solve a protein’s structure, but this and other techniques such as cryogenic electron microscopy are time-consuming, resource-intensive, and costly. For these reasons, current research efforts have turned to computational approaches. In this work, we utilize deep learning technology for predicting energy metrics of proteins with two amino acid insertions or deletions (InDels). The focus of our present work is necessitated by several examples in the literature of diseases that are often caused by double mutations, including hypertrophic cardiomyopathy (Richard *et al.* 2003; Ingles *et al.* 2005). We rely on Rosetta scores to train and test our model because they are widely used, and moreover because there are multiple mentions in the literature of Rosetta scores being validated against experimental ones (Shringari *et al.* 2020).

We extend the RoseNet model for predicting the Rosetta Scores of protein structures (Coffland *et al.* 2023). In addition to using a previously generated dataset of the exhaustive double InDel mutations for three proteins (Turcan *et al.* 2022), we have generated ∼145k mutants each with two InDels for three additional proteins. We show that our methodology can be successfully applied to a wider range of proteins and achieve satisfactory results of Pearson correlation coefficients greater than 0.7 for some Rosetta scores even when our model is trained on a relatively small subset of all possible mutations with double InDels. Our findings show that this technique can be extended to any additional diverse protein.

The mutations in our datasets are generated using a robotics inspired inverse kinematics approach available in the software Rosetta (Leaver-Fay *et al.* 2011). The reliability and accuracy of scores generated by Rosetta have been studied repeatedly (Leman *et al.* 2020; Smith and Meiler 2020) throughout its 20+ years of development and have been repeatedly named among the top tools in proteomics, making these scores a valuable tool in evaluating potential protein mutations. Despite this, users of the Rosetta software are constrained by the time and storage costs of generating comprehensive mutations for a given protein. We advance our previous work (Coffland *et al.* 2023), including the compute pipeline (Turcan *et al.* 2022), for generating the *in silico* mutants for six protein PDB structure files including 1crn, 1csp, 1hhp, 2ckx, 1c44, and 5cvz.

## 2 Related work

Many experiments have been performed analyzing the effects of substitution mutations (Drake and Baltz 1976; Moreira *et al.* 2007; Masso and Vaisman 2008; Pan *et al.* 2022). Such work has been made possible by well-established experimental techniques including alanine scanning and related shotgun approaches which permit high-throughput analyses (Morrison and Weiss 2001) of large datasets of protein variants. Earlier approaches analyzed correlations in mutational behavior between positions in multiple sequence alignments of amino acids to predict contact maps for protein families, and results were compared with crystallographically determined contacts (Gobel *et al.* 1994). Other efforts such as SIMPROT simulate protein sequence evolution (Pang *et al.* 2005) and include comparing InDel-induced structural effects with those from amino acid substitutions (Jilani *et al.* 2022). Despite these and other efforts, van der Flier *et al.* (2024) describe the challenges of predicting the effects of mutations and emphasizes the significance of novel strategies. Recent advances in studying mutant protein structures include RoseTTA fold, a three-track neural network model, but it requires upwards of 30 minutes for a single computational experiment on a 24-GB TITAN RTX GPU (Baek *et al.* 2021).

## 3 Methods

### 3.1 Dataset

We use our previous compute pipeline (Turcan *et al.* 2022) to generate the datasets of double InDel mutants for six proteins (Supplementary Table S1) and use these data for training and evaluating our model. The first three, from previous work (Coffland *et al.* 2023), contain the exhaustive set of all possible mutants with two InDels for PDB structures 1crn, 1csp, and 1hhp. Additionally, we sample the space for all possible mutations with two InDels of three additional proteins by generating random parameter combinations of two insertion positions and two amino acids to be inserted into PDB structures 2ckx, 1c44, and 5cvz (Supplementary Table S1). The additional proteins were randomly chosen for generation from a comprehensive list of all PDB entries that contained the following criteria: proteins are single chained, resolved using X-ray crystallography, and contain no breaks or missing residues. This criterion was established in order to ensure compatibility with previous data generated for this project. For all new proteins, ∼145k new mutants are generated. Due to the varying lengths of these new proteins, the percentage of mutants generated, out of the total possible number of mutants, is inconsistent. We produce ∼3.6% of all possible mutations with two InDels for the protein 5cvz, ∼4.6% for protein 1c44, and ∼10.1% for protein 2ckx. The functions and parameters made use of in Rosetta are detailed in Turcan *et al.* (2022). The availability of our datasets is detailed in Data availability.

For each mutation examined, there are twenty Rosetta Scores (Leaver-Fay *et al.* 2011) associated with each residue, described in Supplementary Table S2. We sum the scores for each residue in order to characterize each mutation by a singular set of twenty Rosetta Scores. Two of the scores are discarded as they are consistent internally for all mutations of a given protein. These scores include yhh_planarity (a special torsional potential to keep the tyrosine hydroxyl in the plane of the aromatic ring) and dslf_fa13 (the disulfide geometry potential). Additionally, insertion position combinations where there is a gap of less than twelve residues between them are discarded due to the difficulty that the software Rosetta has in solving these mutations within the time constraint. We suspect this is related to the inverse kinematics robotics approach used by the software. Prior to training our models, the Rosetta scores for each mutation are z-score normalized with respect to the specific score across all mutations.

### 3.2 RoseNet architecture

The architecture for RoseNet, defined in Coffland *et al.* (2023), is a fully connected neural network. The model, consisting of 1,296,018 parameters, takes as input the two insertion positions and the identities of the two residues being inserted. Each of these discrete values is mapped via an embedding layer to a continuous representation of either position or amino acid, each with an embedding dimension of 200. The embeddings are concatenated and fed through a series of fully connected layers utilizing batch norm (Ioffe and Szegedy 2015), rectified linear unit activation functions Agarap (2019), and skip connections Bishop (1995); Ripley (1996). The output layer is a single linear layer, without an output non-linearity, that maps the last hidden representation to a vector equal in length to the number of scores being predicted. The contents of a single RoseNet block are diagrammed in Supplementary Fig. S1. The architecture for each model consists of a single RoseNet block. Our model is implemented in the Pytorch library (Paszke *et al.* 2019). Although a variety of optimizers, learning rates, and other hyperparameters are considered, we train using the NAdam optimizer (Dozat 2016) with a batch size of 64, a learning rate of $10^{-5}$, and Huber loss (Huber 1964), which is more robust to outliers than the standard squared error loss.

### 3.3 Assessing performance

The performance of RoseNet is measured using the Pearson correlation coefficient. For each protein, we split our data into five datasets. Prior to creating traditional data splits for training, validation, and test, we partition off two extra test sets (Test2 and Test3). Test2 and Test3 are designed to allow us to investigate the ability of the model to generalize to unseen insertion positions, as well as unseen amino acid combinations. The remaining data are split into train, validation, and Test1 randomly at an 80%/10%/10% ratio, respectively. Test2 contains only insertion positions not seen in the train, validation, or Test1 datasets. Test3 contains residue combinations not yet seen in train, validation, or Test1. Test1 acts as a traditional test dataset, unseen during training.

Additionally, we consider the potential difference in performance of the model when inserting residues into different locations of the existing proteins. We categorize the mutant proteins by the insertions into secondary structures, specifically α-helices and β-sheets. For an insertion to be considered a “β-sheet insertion,” at least one of the two residues must be inserted into a β-sheet. Similarly, for inserting into an α-helix, at least one of the two residues being inserted must be into a preexisting α-helix. For a mutation to be categorized as “neither,” both residues must insert into neither an α-helix nor β-sheet.

We also quantify the performance of our model based on insertions into areas that are categorized as low, medium, and high solvent accessible surface areas (SASA). We calculate the SASA as a percentage of the volume size of each residue and categorize these based on their placement relative to the 33^*rd*^ and $66^{th}$ percentiles. Low is categorized as a SASA to volume value less than the 33^*rd*^ percentile, medium as within the 33^*rd*^-66^th^ percentiles, and high is categorized as greater than the 66^*th*^ percentile. Because our datasets are of double InDels, we categorize the insertions as either *low-low* or *high-high* and group everything else into an *other* category. Low-low and high-high refer to both insertions labeled as low or both as high SASA/volume values, respectively, and everything else encompasses the remaining combinations of insertions.

## 4 Results

Tables 1, 2, and 3 display the Pearson correlation coefficient results for each protein across all Rosetta scores for Test1, Test2, and Test3, respectively. Performance metrics for the train and validation sets are provided in Supplementary Figure S2a–f. The protein with the best performance across all test sets is 1hhp, which is the largest protein for which we generated the exhaustive possible mutations with two InDels. In Table 1, proteins 2ckx and 1c44 achieve Pearson correlation coefficients above 0.700 on 9 Rosetta scores. The protein 5cvz, despite having been trained on only ∼3.6% of its possible mutations with double InDels, accomplishes this on two scores. In some cases, the model is capable of outperforming previous results trained on the exhaustive possible mutations with two InDels using only a small fraction of the data. 2ckx, for example, outperforms all previously generated proteins on the Rosetta scores fa_intra_rep and hbond_bb_sc and outperforms the previously generated protein 1crn on 7 out of the total 18 predicted scores.

**Table 1.** Test1 Pearson correlation coefficient results for all proteins.

	Exhaustive^a^			Randomized^b^		
Rosetta score	1crn	1csp	1hhp	2ckx	1c44	5cvz
fa_atr	0.733	0.746	0.723	0.446	0.393	0.410
fa_rep	0.640	0.696	0.690	0.543	0.443	0.473
fa_sol	0.654	0.697	0.781	0.479	0.542	0.443
fa_intra_rep	0.509	0.416	0.430	0.622	0.368	0.318
fa_intra_sol	0.572	0.650	0.771	0.556	0.535	0.511
lk_ball_wtd	0.577	0.482	0.755	0.464	0.377	0.539
fa_elec	0.606	0.745	0.823	0.691	0.743	0.526
pro_close	0.382	0.607	0.581	0.439	0.360	0.278
hbond_sr_bb	0.873	0.611	0.905	0.768	0.756	0.711
hbond_lr_bb	0.884	0.874	0.873	0.700	0.807	0.622
hbond_bb_sc	0.807	0.587	0.808	0.862	0.726	0.779
hbond_sc	0.820	0.471	0.887	0.780	0.762	0.229
omega	0.710	0.599	0.801	0.401	0.753	0.340
fa_dun	0.892	0.847	0.870	0.798	0.676	0.502
p_aa_pp	0.486	0.812	0.745	0.613	0.757	0.480
ref	0.999	1.000	1.000	0.997	0.997	0.997
rama_prepro	0.617	0.742	0.659	0.629	0.778	0.466
total	0.647	0.700	0.701	0.545	0.452	0.475

a These proteins had their exhaustive possible mutations generated. The Test1 dataset consists of 22,387 mutants for 1crn, 57,517 mutants for 1csp, and 137,958 mutants for 1hhp.

b These proteins had a random subset of ∼145k mutants generated. The Test1 dataset consists of 13,791 mutants for 2ckx, 13,514 mutants for 1c44, and 13,959 mutants for 5cvz.

The best performance for all proteins is on the Rosetta score “ref,” for which all proteins achieve at least 0.996 Pearson across all test sets. This is not surprising, as the “ref” term is only dependent on the sequence. For Test1 in Table 1, the scores with the second highest correlation coefficients are hbond_lr_bb (1csp, 1crn, and 1c44), hbond_sr_bb (1hhp), and hbond_bb_sc (2ckx and 5cvz). Each of these Rosetta scores is related to hydrogen bond energies. The scores with the lowest correlation coefficients are fa_intra_rep (1hhp, 1csp, and 1c44), pro_close (1crn), omega (2ckx), and hbond_sc (5cvz). Interestingly, from among these Rosetta scores that yielded the lowest correlations, three of them are penalty metrics, and as such do not pertain to a measure of the quality of a score to capture the effect of a mutation. Table 1 shows the results for Test1. We find the model to perform better on Test1 than Test2 or Test3. We also find that the model demonstrates superior performance on Test1 and Test3 (shown in Table 3), which measures the performance of the model on previously unseen amino acid combinations, compared to Test2, which measures the performance of the model on mutants with unseen insertion position combinations, as shown in Table 2. This trend is consistent with the results found in previous work (Coffland *et al.* 2023).

**Table 2.** Test2 Pearson correlation coefficient results for all proteins, showing performance on previously unseen insertion positions.

	Exhaustive^a^			Randomized^b^		
Rosetta score	1crn	1csp	1hhp	2ckx	1c44	5cvz
fa_atr	0.594	0.722	0.691	0.189	0.297	0.155
fa_rep	0.603	0.714	0.657	0.280	0.087	0.362
fa_sol	0.580	0.666	0.765	0.201	0.362	0.242
fa_intra_rep	0.483	0.438	0.405	0.337	0.136	0.146
fa_intra_sol	0.573	0.671	0.778	0.710	0.324	0.059
lk_ball_wtd	0.538	0.416	0.738	−0.040	0.412	0.216
fa_elec	0.459	0.703	0.768	0.495	0.727	0.261
pro_close	0.387	0.665	0.572	0.211	0.256	0.205
hbond_sr_bb	0.813	0.649	0.868	0.551	0.599	0.553
hbond_lr_bb	0.760	0.846	0.830	0.476	0.538	0.229
hbond_bb_sc	0.767	0.572	0.819	0.588	0.126	0.565
hbond_sc	0.755	0.344	0.865	0.534	0.390	0.108
omega	0.591	0.552	0.766	−0.118	0.631	0.062
fa_dun	0.892	0.870	0.857	0.661	0.631	0.386
p_aa_pp	0.507	0.780	0.740	0.279	0.709	0.208
ref	0.999	0.999	1.000	0.997	0.998	0.997
rama_prepro	0.597	0.606	0.645	0.072	0.756	0.275
total	0.604	0.719	0.667	0.305	0.103	0.365

a These proteins had their exhaustive possible mutations generated. The Test2 dataset consists of 12 722 mutants for 1crn, 32 392 mutants for 1csp, and 77 447 mutants for 1hhp.

b These proteins had a random subset of ∼145k mutations generated. The Test2 dataset consists of 3,928 mutants for 2ckx, 3926 mutants for 1c44, and 4181 mutants for 5cvz.

**Table 3.** Test3 Pearson correlation coefficient results for all proteins, showing performance on previously unseen amino acid combinations.

	Exhaustive^a^			Randomized^b^		
Rosetta Score	1crn	1csp	1hhp	2ckx	1c44	5cvz
fa_atr	0.732	0.741	0.714	0.442	0.377	0.410
fa_rep	0.640	0.705	0.695	0.501	0.375	0.472
fa_sol	0.647	0.694	0.776	0.484	0.502	0.450
fa_intra_rep	0.538	0.414	0.436	0.592	0.384	0.298
fa_intra_sol	0.547	0.668	0.762	0.581	0.529	0.468
lk_ball_wtd	0.601	0.485	0.754	0.430	0.398	0.530
fa_elec	0.609	0.740	0.824	0.689	0.737	0.530
pro_close	0.387	0.615	0.578	0.390	0.313	0.185
hbond_sr_bb	0.872	0.610	0.903	0.757	0.737	0.702
hbond_lr_bb	0.878	0.873	0.873	0.716	0.801	0.630
hbond_bb_sc	0.802	0.585	0.812	0.833	0.709	0.775
hbond_sc	0.817	0.450	0.889	0.785	0.762	0.251
omega	0.705	0.593	0.797	0.342	0.760	0.336
fa_dun	0.895	0.848	0.863	0.811	0.688	0.470
p_aa_pp	0.502	0.816	0.743	0.596	0.740	0.465
ref	0.999	0.999	1.000	0.997	0.996	0.996
rama_prepro	0.635	0.740	0.650	0.563	0.770	0.434
total	0.648	0.710	0.707	0.503	0.384	0.473

a These proteins had their exhaustive possible mutations generated. The Test3 dataset consists of 12 432 mutants for 1crn, 31 895 mutants for 1csp, and 76 336 mutants for 1hhp.

b These proteins had a random subset of ∼145k mutations generated. The Test3 dataset consists of 3631 mutants for 2ckx, 3892 mutants for 1c44, and 3848 mutants for 5cvz.

**Figure 1.**
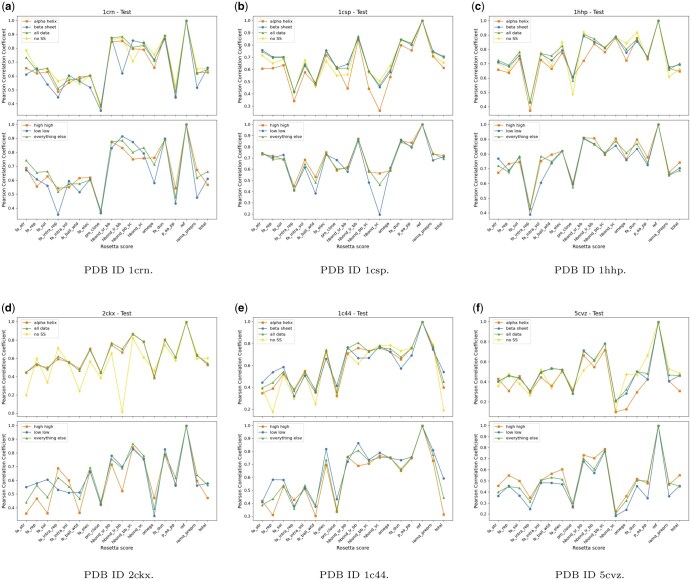
Plotting the secondary structures (top panels in a–f) where at least one insertion is into an α-helix or β-sheet, no insertions into a secondary structure, and all data. Per-residue SASA (bottom panels in a–f) where the SASA/volume of both insertions are <=33rd percentile (low-low), >66th percentile (high-high), and every other combination (everything else) on Test1.

We measure the performance of RoseNet separated by insertions into α-helices, β-sheets, and insertions not into secondary structures. We find the highest accuracy of predicting Rosetta scores for new mutations is generally associated with insertions into β-sheets, whereas the lowest accuracy is associated with insertions into α−helices. These results are shown in Fig. 1a–f plotting the Pearson correlation coefficients across Rosetta scores per secondary structure category (top) for insertions into α−helices (orange, square), β-sheets (blue, circle), all data (green, triangle), and no secondary structure (yellow, diamond). This effect becomes more pronounced as general prediction performance increases. This trend is especially apparent in the Rosetta score “total,” which is calculated using Rosetta’s all-atom energy function to produce a single scalar value representing the overall stability and physical plausibility of the structure (Alford *et al.* 2017). These results are intuitively logical, as α-helices are known to have a greater effect on the overall structure of a protein, and thus changing them is akin to making changes to the structurally stabilizing elements of a protein. This has a high likelihood of resulting in impactful InDel pairs of mutations, which is more difficult to predict than if insertions are made into elements of a protein that are not secondary structures.

We find a similar pattern when plotting the SASA values. Figure 1 plots the Pearson correlation coefficients across Rosetta scores per SASA category (bottom) for high-high (orange, square), low-low (blue, circle), and everything else (green, triangle). When the insertion positions of a given mutant fall in the category of low-low, these results show that the model displays poorer performance than insertions into high-high. When the insertion positions of a given mutant fall in the category of high-high, the model is better able to predict the energy metrics for those mutants, and this pattern also degrades as performance declines. Residues with low SASA values are considered buried within the protein structure. These residues are more sensitive to changes from mutations due to their impact on protein structure. Therefore, the model encounters greater difficulty in accurately predicting the energy metrics of these mutants. These results are naturally plausible, as insertions represented by the low-low category are insertions into solvent inaccessible regions of a protein, which for most globular proteins are core residues that present many more contact points with neighboring residues than do residues with high SASA values. Inserting residues into the core of a protein not only introduces steric clashes in the immediate vicinities of the insertion points, but also has a type of propagation effect that may introduce steric clashes of far-away residues.

## 5 Discussion

We extend the RoseNet architecture for use on an additional three proteins, in addition to the proteins modeled in previous work. Through training on a small fraction of the number of datapoints previously used, described in Sec. Methods, we observe comparable performance to the results from our previous work (Coffland *et al.* 2023), highlighted in Section 3. We also find evidence to support previous conclusions, specifically that performance on Test3 is generally higher than performance on Test2, indicating that the models are typically capable of generalizing to previously unseen residue combinations better than to previously unseen insertion positions.

By conducting experiments training on fewer datapoints, segregating out additional test sets, and varying the lengths of the proteins studied, we seek to evaluate our model’s robustness to a lack of training data and pinpoint the features of training data that need to be represented in a dataset in order to achieve satisfactory performance with a given model.

We analyze the performance of our model by predicting energy metrics for previously unseen insertion positions, previously unseen residue combinations, insertions into secondary structures, and insertions into areas of high and low SASA as a percentage of the volume size of each residue for all six proteins. We find our models frequently achieve higher performance when inserting into β-sheets and unstructured areas of the protein, as opposed to α-helices. We also find that our models typically perform better when inserting into areas of high SASA.

Our extension of RoseNet demonstrates its ability to handle novel protein structures, and our methodology can be extended to any additional protein. For three new proteins, we achieve satisfactory results of Pearson correlation coefficients greater than 0.7 for many Rosetta scores despite training on a small subset of the exhaustive mutants. We also uncover further information regarding the number of datapoints needed in order to accurately model the energy metrics of each protein. We noticed that not all Rosetta scores are equally sensitive to the effects of double insertion mutations. Identifying those that are most sensitive or that can predict the effects of double InDels is left to future work. Distributions for all scores across all mutations for all proteins can be found in Supplementary Tables S3 and S4. Future experimentation also includes implementing a protein agnostic model that is trained across various proteins and takes as input the entire amino acid sequence to predict its Rosetta scores. We also plan to train and evaluate a model to predict the energy metrics of each residue in the sequence.

## Supplementary Material

vbae198_Supplementary_Data

## Data Availability

The code used for training and evaluating the models in the study and the data underlying this article are available at https://github.com/hutchresearch/RoseNet.
