# Supplementary material for: Energy metric prediction for double insertion mutants via the RoseNet deep learning framework
Source: Bioinform Adv. 2025 Jan 2;5(1):vbae198. doi: 10.1093/bioadv/vbae198 (PMC12133284; doi:10.1093/bioadv/vbae198)
Supplement: vbae198_Supplementary_Data [file vbae198_supplementary_data.pdf]

Supplement to Energy Metric Prediction for Double Insertion Mutations via the RoseNet Deep Learning Framework

Sarah Coffland, Katie Christensen, Brian Hutchinson, Filip Jagodzinski

Supplementary Materials

Additional materials are provided here showing the distributions of all Rosetta scores for all mutants across all proteins, as well as a diagram of the model architecture as it is used in this work.

Table S 1. Proteins, PDB IDs, wild type protein lengths, and total number of mutants in their train, validation, and test sets.

PDB ID	Protein	Length	Number Mutants
1crn	Cambrin	46	249020
1csp	Bacillus Subtilis Major Cold Shock	67	639457
2ckx	Telometric binding factor from Nicotiana tabacum	83	145461
1hhp	HIV-1 isolate BRU	99	1533354
1c44	Sterol carrier protein 2	123	142952
5cvz	Satellite panicum mosaic virus	141	147613

Table S 2. Rosetta energy and related metric scores **?**. e=energy; res=residue; B=backbone; SC=side chair; LJ=Lennard-Jones

Rosetta score/metric	Meaning
fa_atr	LJ attractive force between atoms in different res
fa_rep	LJ repulsive force between atoms in different res
fa_sol	Lazaridis-Karplus solvation e
fa_intra_sol_xover4	Intra-residue Lazaridis-Karplus solvation e
lk_ball_wtd	Asymmetric solvation e
fa_intra_rep	LJ repulsive between sam res atoms
fa_elec	Coulombic electrostatic potential with distance-dependent dielectric
pro_close	Proline ring closure e and e of ψ angle of preceding res
hbond_sr_bb	B-B hbonds close in primary sequence
hbond_lr_bb	B-B hbonds distant in primary sequence
hbond_bb_sc	SC-B hbond e
hbond_sc	SC-SC hbond e
dslf_fa13	Disulfide geometry potential
rama_prepro	Ramachandran preferences
omega	Omega dihedral in the backbone
p_aa_pp	Probability of amino acid, given torsion values for ϕ and ψ
fa_dun	Internal energy of SC rotamers
yhh_planarity	Torsional potential to keep tyrosine hydroxyl in plane of aromatic ring
dslf_fa13	Disulfide geometry potential
ref	Reference e for each amino acid
total	The total weighted score

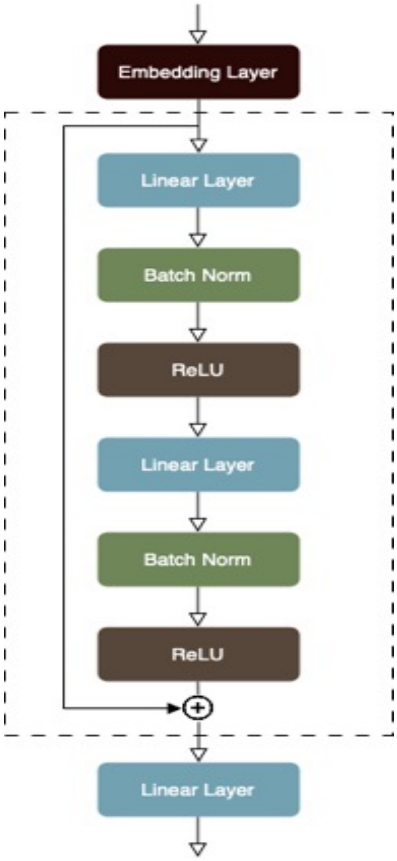

Fig. S 1. Architecture of a single RoseNet block.

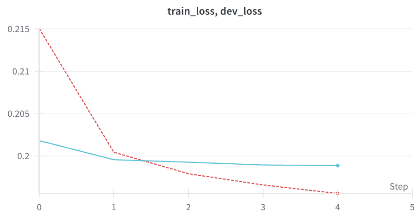

(a) PDB ID 1crn.

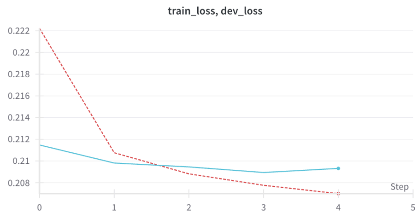

(b) PDB ID 1csp.

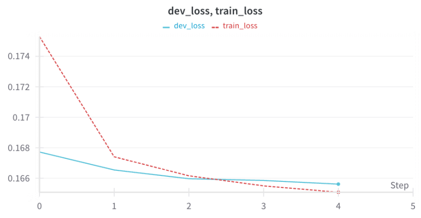

(c) PDB ID 1hhp.

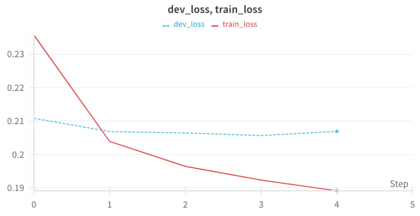

(d) PDB ID 2ckx.

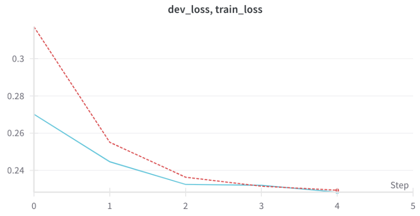

(e) PDB ID 1c44.

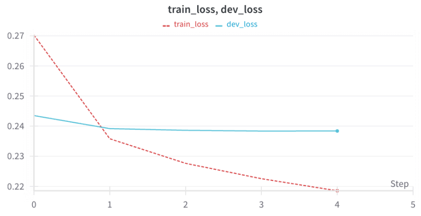

(f) PDB ID 5cvz.

Fig. S 2. Train and validation loss curves for each protein. One step is equivalent to one epoch.

Table S 3. Metrics for proteins 1crn, 1csp, and 1hhp.

	1crn Metrics			
Scores	Min	Max	Avg	Std
fa_atr	-614.776	-330.004	-437.536	36.144
fa_rep	40.419	21748.721	1209.477	1308.177
fa_sol	227.778	428.352	293.463	18.654
fa_intra_rep	0.738	3.663	0.927	0.112
fa_intra_sol	9.606	22.394	13.268	1.180
lk_ball_wtd	-24.353	0.796	-12.720	2.399
fa_elec	-134.736	-23.629	-79.824	11.391
pro_close	2.556	1418.418	82.256	117.945
hbond_sr_bb	-25.750	-3.586	-14.370	2.917
hbond_lr_bb	-11.606	1.000	-2.838	2.401
hbond_bb_sc	-19.475	1.000	-5.756	2.640
hbond_sc	-11.472	1.000	-0.632	1.807
omega	-1.142	33.170	5.762	2.377
fa_dun	70.727	275.460	121.807	16.049
p_aa_pp	-23.379	53.244	-0.892	7.257
ref	68.557	92.474	81.079	4.691
rama_prepro	-11.876	165.707	36.738	19.373
total	-13.203	22187.982	1277.327	1332.145
	1csp Metrics			
Scores	Min	Max	Avg	Std
fa_atr	-864.704	-368.446	-617.148	37.836
fa_rep	74.553	27730.188	2081.462	2272.057
fa_sol	267.168	529.968	360.359	24.818
fa_intra_rep	0.892	5.747	1.699	0.140
fa_intra_sol	15.136	39.672	27.491	1.733
lk_ball_wtd	-26.982	1.848	-13.588	2.785
fa_elec	-178.533	8.901	-106.738	18.846
pro_close	1.256	1040.832	45.219	80.596
hbond_sr_bb	-17.181	-0.441	-3.269	1.136
hbond_lr_bb	-29.944	0.030	-17.703	3.917
hbond_bb_sc	-20.479	1.000	-3.610	2.143
hbond_sc	-11.034	1.000	0.245	1.241
omega	-1.238	44.958	4.358	2.529
fa_dun	142.391	681.676	395.794	39.389
p_aa_pp	-41.020	35.729	-16.669	9.600
ref	8.997	32.915	21.529	4.690
rama_prepro	-10.609	136.021	27.296	17.286
total	12.170	27893.127	2171.773	2296.951
	1hhp Metrics			
Scores	Min	Max	Avg	Std
fa_atr	-1173.711	-775.846	-944.329	35.736
fa_rep	170.410	29851.590	2597.535	2365.771
fa_sol	408.194	717.789	514.166	26.873
fa_intra_rep	2.739	8.392	3.160	0.212
fa_intra_sol	31.513	49.655	39.444	1.811
lk_ball_wtd	-27.978	7.579	-9.908	3.752
fa_elec	-245.556	-40.007	-162.118	21.992
pro_close	6.423	1671.722	117.626	138.957
hbond_sr_bb	-22.194	-1.922	-8.663	2.369
hbond_lr_bb	-34.004	-10.367	-23.465	3.412
hbond_bb_sc	-25.499	0.909	-9.267	2.890
hbond_sc	-16.842	0.618	-5.823	2.333
omega	20.967	86.129	33.702	3.794
fa_dun	521.786	957.194	622.484	36.373
p_aa_pp	-49.238	55.025	-21.452	9.894
ref	108.798	132.715	121.331	4.691
rama_prepro	10.950	202.910	51.532	19.552
total	344.157	30287.383	2901.001	2416.587

[1] All values are rounded to the nearest significant figure.

Table S 4. Metrics for proteins 2ckx, 5cvz, and 1c44.

	2ckx Metrics			
Scores	Min	Max	Avg	Std
fa_atr	-1716.806	-808.586	-1071.120	42.072
fa_rep	155.922	85976.797	2929.860	2874.278
fa_sol	620.822	1183.394	717.797	23.799
fa_intra_rep	2.060	5.351	2.503	0.190
fa_intra_sol	40.527	58.765	47.817	1.942
lk_ball_wtd	-28.826	1.459	-14.083	3.282
fa_elec	-312.052	-119.597	-242.817	19.291
pro_close	1.717	1187.924	41.759	93.350
hbond_sr_bb	-54.082	-28.371	-40.820	3.608
hbond_lr_bb	-7.813	1.000	-0.232	1.395
hbond_bb_sc	-28.942	-2.593	-14.951	4.606
hbond_sc	-27.382	-0.870	-13.390	4.510
omega	-2.922	35.501	2.042	2.272
fa_dun	258.634	675.706	389.755	42.254
p_aa_pp	-30.406	30.355	-14.812	6.580
ref	31.120	55.038	43.669	4.696
rama_prepro	-26.534	122.931	12.142	16.555
total	-138.857	85692.344	2760.165	2882.805
	5cvz Metrics			
Scores	Min	Max	Avg	Std
fa_atr	-1900.392	-721.568	-1435.766	62.729
fa_rep	243.304	83141.719	2906.540	3325.507
fa_sol	428.730	1052.206	832.447	34.171
fa_intra_rep	3.178	7.854	3.596	0.183
fa_intra_sol	42.727	59.858	49.165	1.656
lk_ball_wtd	-47.904	-2.726	-22.549	3.926
fa_elec	-363.052	-24.956	-284.001	27.887
pro_close	5.641	1254.712	63.660	102.281
hbond_sr_bb	-17.136	-3.517	-8.675	1.471
hbond_lr_bb	-57.671	1.000	-46.749	6.030
hbond_bb_sc	-49.608	-5.265	-28.764	5.497
hbond_sc	-15.935	1.000	-0.499	1.536
omega	-4.055	40.708	1.835	2.535
fa_dun	529.718	876.596	616.074	27.814
p_aa_pp	-49.206	27.216	-23.568	8.182
ref	140.202	164.120	152.721	4.701
rama_prepro	18.048	169.109	65.269	17.371
total	49.567	83220.383	2825.784	3351.344
	1c44 Metrics			
Scores	Min	Max	Avg	Std
fa_atr	-2469.216	-976.756	-1358.978	49.393
fa_rep	279.772	158637.969	3161.592	3105.271
fa_sol	709.206	1677.945	886.823	29.121
fa_intra_rep	2.493	6.528	2.799	0.169
fa_intra_sol	41.647	58.036	47.963	1.678
lk_ball_wtd	-50.115	-3.049	-21.765	3.222
fa_elec	-401.056	-95.429	-315.244	28.414
pro_close	2.808	1188.311	43.080	97.534
hbond_sr_bb	-51.673	-25.245	-37.083	2.951
hbond_lr_bb	-35.081	-0.602	-24.388	4.680
hbond_bb_sc	-37.109	-6.036	-23.537	3.902
hbond_sc	-16.182	0.755	-5.999	2.001
omega	13.204	67.565	30.589	4.922
fa_dun	451.146	838.892	552.830	30.098
p_aa_pp	-53.735	41.761	-19.824	10.057
ref	51.175	75.092	63.687	4.701
rama_prepro	-13.160	187.958	75.520	23.679
total	-76.235	158467.766	3043.111	3123.937

[1] All values are rounded to the nearest significant figure.
